# Relationship Between Ultrasound Diagnosis, Symptoms and Pain Scale Score on Examination in Patients with Uterosacral Ligament Endometriosis

**DOI:** 10.3390/jcm13226901

**Published:** 2024-11-16

**Authors:** Shae Maple, Eva Bezak, K. Jane Chalmers, Nayana Parange

**Affiliations:** Allied Health and Human Performance, University of South Australia, GPO Box 2471, Adelaide, SA 5001, Australia

**Keywords:** deep infiltrating endometriosis, gynecology, sonography, site-specific tenderness, transvaginal ultrasound, uterosacral ligaments

## Abstract

**Background/Objectives:** This study investigated patient pain descriptors for transvaginal ultrasound (TVS) diagnostic evaluation of endometriosis for uterosacral ligaments (USLs), including correlation between USL thickness and site-specific tenderness (SST). It further investigated if SST could positively assist diagnosing endometriosis on TVS. **Methods:** TVS images and SST pain descriptors were collected from 42 patients. SST was evaluated by applying sonopalpation during TVS. The images were presented to six observers for diagnosis based on established USL criteria. Following this, they were given the SST pain scores and asked to reevaluate their diagnosis to assess if the pain scores impacted their decision. **Results:** An independent *t*-test showed that the patients with an endometriosis history had higher pain scores overall (7.2 ± 0.59) compared to the patients with no history (0.34 ± 0.12), t (40) = 8.8673. Spearman’s correlation showed a strong correlation to the pain scale score for clinical symptoms (*r* = 0.74), endometriosis diagnosis (*r* = 0.78), USL thickness (*r* = 0.74), and when USL nodules were identified (*r* = 0.70). Paired *t*-tests showed that the observers demonstrated a higher ability to correctly identify endometriosis with the pain scale information (33 ± 8.83) as opposed to not having this information (29.67 ± 6.31), which was a statistically significant change of 3.33, t (5) = 2.7735. **Conclusions:** Patients with an endometriosis history have significantly higher pain scores on TVS compared to patients with no endometriosis history. A strong correlation was shown between SST pain scores and patient symptoms, USL thickness, and USL nodules. Inclusion of SST alongside TVS imaging shows promise, with these results demonstrating a higher ability to diagnose endometriosis with additional SST pain scale information.

## 1. Introduction

Endometriosis occurs when endometrial-like tissue extends outside of the uterine cavity [1], with the subtype of deep endometriosis (DE) occurring when endometriotic tissue infiltrates >5 mm under the peritoneum [2,3,4,5]. So far, the mechanisms of endometriosis have not been fully agreed upon, but endometriosis is considered a chronic inflammatory process associated with immune processes [3] that results in fibrosis and organ immobility [6] that cause chronic pelvic pain [1,3,6]. This impacts physical and mental health, as well as the social well-being of the patient, due to a significant deterioration in the quality of life [3]. It is estimated that endometriosis affects 10% of female-born people [1]; however, the true frequency of endometriosis is uncertain, because a definitive diagnosis requires laparoscopy and histopathology, which is not always accessible. With the new acknowledgment that transvaginal ultrasound (TVS) is capable of and accurate in detecting endometriosis and with the current recognition of diagnostic delays, as well as the invasiveness, cost, and post-surgical recovery time of surgery, the European Society of Human Reproduction and Embryology (2022) guidelines [7] now recommend the use of TVS as the first-line imaging tool for endometriosis.

In 2016, the International Deep Endometriosis Analysis group (IDEA) [8] developed the first guidelines for reproducible sonographic features of endometriosis, with TVS now demonstrating reliable accuracy for diagnosing DE [9]. The most current International Consensus Statement (2024) [10] advises that (a) “TVS performed by adequately trained operators is recommended as the first-line imaging tool” [10] and (b) “imaging with TVS can help to preoperatively predict the presence of DE of the USLs” [10], although ongoing research is still needed to support this statement. As such, diagnosis of DE of the uterosacral ligaments (USLs) remains the most difficult [10,11,12,13]. Studies have shown improvements by implementing the standardized posterior approach [14], providing evidence that with refinement of these techniques and ultrasound criteria, diagnostic ability can continue to improve [15,16].

Broadly speaking, endometriosis has a wide range of symptoms, including chronic pelvic pain [2,17], dysmenorrhea [2,5,18,19,20,21,22], dyspareunia [18,20,22,23,24,25,26], and/or infertility [6,14]. However, the relationship between the type and site of the endometriotic lesions and the severity of pelvic symptoms is a matter of debate, as data from numerous studies on such relationships are contradictory [18]. There has been, however, a strong association observed in several studies between deep posterior compartment DE deposits and dyspareunia [18,20,22,23,24,25].

USLs sit in this posterior compartment and can be easily visualized with TVS [10,12]. USLs are also one of the most common sites of DE [4,6,10,14,27], as the distal section attaches to the posterior cervix at the torus uterinus (TU) [8,14,28]. This anatomical location allows USLs to also be associated with rectal DE [6,15] and adhesions to the ovary in the presence of an endometrioma, often visualized with one or both ovaries located posteriorly at the TU, all contributing to dyspareunia and pelvic pain [6,29,30]. Therefore, there is some benefit in including tenderness-directed TVS scanning [31].

These pain symptoms associated with endometriosis can be replicated by site-specific tenderness (SST) ultrasound scanning by using gentle transductor pressure. Pain scale tests can be used to measure SST and provide valuable information on the specific pain experienced by endometriosis sufferers [32]. Incorporating SST over the USLs and the relationship with endometriosis is a valuable component of the TVS examination, providing beneficial insight into the extent of endometriosis [33], as well as reliably assisting in the diagnosis of posterior compartment DE [34,35,36]. Recent reviews [12] have determined that more refinement is still needed for incorporating SST to assist in diagnosing DE, as current literature has little guidance on pain scale correlation of USL changes in the diagnosis of DE, such as using a visual analogue or numerical rating scale to capture the severity of pain during TVS [12]. Without this information, it is difficult to determine the correlation of SST with endometriotic nodules and therefore difficult to determine the true value of SST-TVS of the USLs.

In this light, the aim of this study was to evaluate patient pain descriptors in the ultrasound diagnostic evaluation of endometriosis of the uterosacral ligaments and investigate if SST descriptors can positively assist in diagnosing endometriosis on TVS.

## 2. Methods

### 2.1. Study Design and Ethics

This observational cohort study was conducted between November 2021 and September 2022. Methodology followed the Strengthening the Reporting of Observational studies in Epidemiology (STROBE) guidelines [37]. This study was approved by the University of South Australia Human Research Ethics Committee (project number 204278). Written informed consent was obtained from all subjects before the study.

### 2.2. Study Setting and Patient Participants

Prior to patient recruitment, sample size calculation was performed using G*Power software version 3.1.9.7 [38], which estimated a minimum sample size of 42 patients with five observers to show significant results with a large effect size of 95%, a power of 0.8, and a 95% confidence interval.

**Figure 1:**
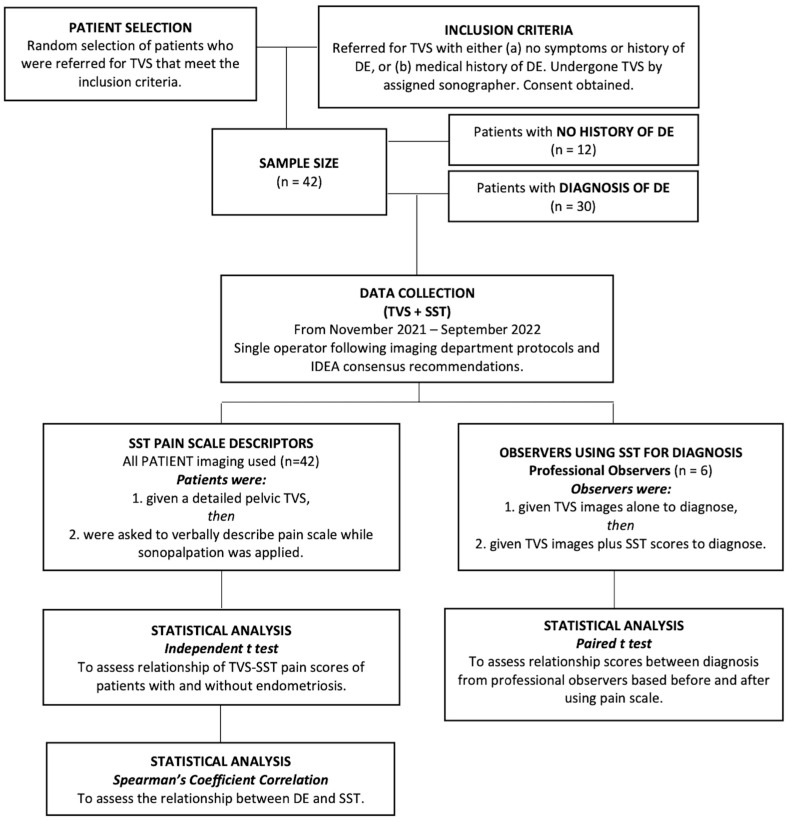
Flowchart summarizing the methodology process and the inclusion criteria of eligible patients and observers in the study. TVS, transvaginal ultrasound, DE, Deep Endometriosis, IDEA, International Deep Endometriosis Analysis, SST, site-specific tenderness.

Forty-two (42) patients who met the inclusion criteria (Figure 1) and who presented to an outpatient general imaging private practice in South Australia following a referral from either a general practitioner or a gynecologist from November 2021 until September 2022 were included in this study. Twelve (12) of the patients presented with no history or symptoms of endometriosis, while 30 patients presented with medically diagnosed endometriosis, either via laparoscopy or clinically diagnosed by a gynecologist.

Detailed TVS for endometriosis was performed, and the patient pain descriptors were collected. Analysis of the ultrasound characteristics of the USLs was performed by six observers, including five Accredited Medical Sonographers as per the Australia Sonographers Accreditation Registry (ASAR) and one Royal Australian and New Zealand College of Obstetricians and Gynaecologists (RANZCOG)-accredited radiologist. A multidisciplinary approach was achieved by selecting observers with different levels of experience and qualifications to reduce professional bias and reflect practice in clinical settings. The detailed process is shown in Figure 1.

### 2.3. Study Objectives

The first objective was to evaluate the use of patient pain descriptors in the ultrasound diagnostic evaluation of endometriosis, including the correlation between USL thickness and SST. The second objective was to investigate if this additional patient SST pain score information altered the diagnostic decision of endometriosis by imaging professionals when diagnosing endometriosis on TVS.

### 2.4. Outcome Measures

The ultrasound images were presented to the observers for diagnosis. Following this, they were given the SST pain scores for each patient participant and asked to reevaluate their diagnosis to assess if the pain score impacted their decision.

### 2.5. Data Sources and Collection

#### 2.5.1. Image Acquisition

To reduce variability in the acquired images, TVS was performed by an experienced accredited sonographer with 15 years’ experience using Canon i800, Aplio 500, Aplio a550, and Aplio XG (Canon Medical System Corporation, Otawara, Tochigi, Japan) ultrasound machines with an 11CV3, 11CV, or 9C3 (3–11 MHz) transvaginal transducer.

Assessment of the complete pelvis was performed to include the uterus, cervix, adnexa, and ovaries, as well as the vagina, RVS, urinary bladder, rectosigmoid, and the USLs. Each examination was performed as per the referral request and adhered to general practice imaging protocols congruent with the national Australian Society of Ultrasound in Medicine guidelines [39] and the recommended four-step method outlined within the IDEA consensus [8,40] for the diagnosis of endometriosis.

To ensure consistency and reproducibility, the current recommended ultrasound techniques to visualize the USLs [12,14] were used. This involved placing the transducer into the posterior vaginal fornix and withdrawing it backwards into the vagina. Keeping the transducer in the longitudinal plane, it was then angled inferolaterally to visualize the cervix and then rotated approximately 45° until a hyperechoic line, which is the USL, came into view [12,14]. Findings of endometriosis were diagnosed according to the systematic approach described by the IDEA [8] group.

#### 2.5.2. Assessment of Pain During TVS for Endometriosis

SST was evaluated by applying sonopalpation during TVS. The patients were asked to verbally describe their SST with pain during the scan in line with the validated short-form McGill Pain Scale questionnaire (SF-MPQ) [41,42]. The SF-MPQ comprises two measurement components: the Pain Rating Index (PRI) and the Present Pain Intensity (PPI) visual analogue scale. For *PRI*, the patients were asked to describe what the pain felt like with six qualitative reports of pain: bloating (0), tender (1), dull ache (2), cramping or pressure (3), sharp (4), or stabbing (5). For PPI, the patients were asked “Can you tell me how painful this spot is on a scale of 0–10 where 0 represents no pain and 10 represents the worst pain imaginable”. The sum of the sensory and affective components produced the McGill total pain score [32].

#### 2.5.3. Observers

Each observer reviewed the 42 cases of USLs both with and without an endometriosis history. In each case, the reviewed images included a still image of the USL in the longitudinal plane and a video clip demonstrating a sweep through and/or some real-time dynamics of the USL in question. The observers were blinded to the group of patients and each other’s responses.

Normal USLs appeared as a smooth linear, hyperechoic, and homogeneous structure that slid freely over the vaginal wall when transducer pressure was applied and released [12]. USLs were considered to have endometriotic involvement when they appeared thickened with some heterogenous changes and/or when there was hypoechoic linear thickening with regular or irregular margins of the USL. Endometriotic nodules were visualized as a solid hypoechoic nodule with regular, irregular, or stellate margins that were tender on sonopalpation and fixed to the surrounding pelvic structures [12].

The observers were asked to make a diagnosis of endometriosis based on the USL images alone, with a follow-up question where they were given the documented results from the SST evaluation based on the SF-MPQ pain scale grading [32,41,42] to see if the pain scale information influenced their response to the characteristics they were observing. Images and data analysis for this paper were compiled for offline review using Qualtrics software, version XM, June 2023, (Qualtrics, 2023, Provo, UT, USA).

### 2.6. Statistical Analyses

Descriptive statistics for patient participants’ demographics were recorded and collated for analysis. Ultrasound evaluation of the patient pain descriptors for endometriosis was analyzed using independent sample *t*-tests [43] on the sample of 42 patients to determine differences between the SF-MPQ SST pain score grading in patients with endometriosis and those without. SST pain scale (0–15) was calculated as a mean ± SD. Spearman’s correlation coefficient [43] was used to determine the relationship between endometriosis characteristics on TVS and SST pain scale grading, where the outcome results were interpreted according to the degree of association as strong (*r* = 0.7–1), moderate (*r* = 0.5–0.7), or low (*r* = 0.3–0.5) after taking significant correlation (*p* < 0.01 or *p* < 0.05) values into consideration. Paired *t*-tests [43] were used on the six professional observer results to determine whether there was a statistically significant relationship between the image interpretation of USLs without pain scale information compared to the image interpretation of USLs with pain scale information. Results were considered statistically significant when *p* < 0.05. Statistical analyses were performed with Stata/SE 18.0 for Mac (Intel 64-bit), revision 4 October 2023 [43].

## 3. Results

### 3.1. Patient Participant Characteristics and Ultrasound Data Sets

Forty-two (42) women were included in the study. The mean age was 33.6 (range 18–54) years. Four of the women in our final analysis aged over 50 years had chronic known endometriosis. The presenting symptoms were dysmenorrhea in 16/42 (38.1%), chronic pelvic pain in 26/42 (61.9%), dyspareunia in 5/42 (11.9%), and infertility in 6/42 (14.3%). At laparoscopy, 10/42 (23.8%) women had confirmed endometriosis, 7/42 (16.7%) were clinically diagnosed, while 13/42 (31%) were referred for TVS with clinical suspicion of endometriosis. Table 1 details the findings of endometriosis on ultrasound for the patients with a known or suspected history of endometriosis. Of the 30 women with known endometriosis, 3/30 (10%) women had endometriosis in a single location (excluding USL changes), whilst the remaining 90% had endometriosis in two or more locations. This included focal endometriotic lesions including rectosigmoid, rectocervical, and focal LSCS scar lesions 21/30 (70%), medialized ovaries 9/30 (30%), adherent ovaries 21/30 (70%), endometrioma (14/30 (46.7%), a fixed or rotated uterus 11/30 (36.7%), and obliterated POD 4/30 (13.3%). Also, 19/30 (63.3%) of participants had evidence of adenomyosis.

There was no DE present in any participant who presented with no symptoms or history of DE. Of the 30 participants with known DE, 12 (40%) USL nodules were detected, and all 30 appeared thickened >3 mm. Measurements of the USLs were taken and recorded for all participants.

### 3.2. Pain Descriptors and Endometriosis Relationship

There was a broad range of reported SF-MPQ total pain scores, ranging from 0 to 15, which are shown in detail in Table 2. The independent *t*-test results showed that the patients with an endometriosis history had statistically significantly higher pain scores overall (7.2 ± 0.59) compared to the patients with no history of endometriosis (0.34 ± 0.12), t (40) = 8.8673, *p* = 0.000. Furthermore, the results showed that the patients with an endometriosis history had statistically significantly higher pain scores specific to the USLs (8.15 ± 0.54) compared to the patients with no history of endometriosis (0.21 ± 0.21), t (82) = 9.1645. Patients with USL nodules demonstrated a further increase in pain scores (12.8 ± 0.38) compared to the patients with USLs with DE features but no identified nodule (7.4 ± 0.92), t (28) = 4.3366.

Spearman’s correlation was used to assess the relationship between TVS endometriosis characteristics and pain scale in 42 participants. For the analysis of USL nodules, 30 participants with diagnosed endometriosis were included to compare pain levels between those with and without a focal USL nodule.

**Figure 2:**
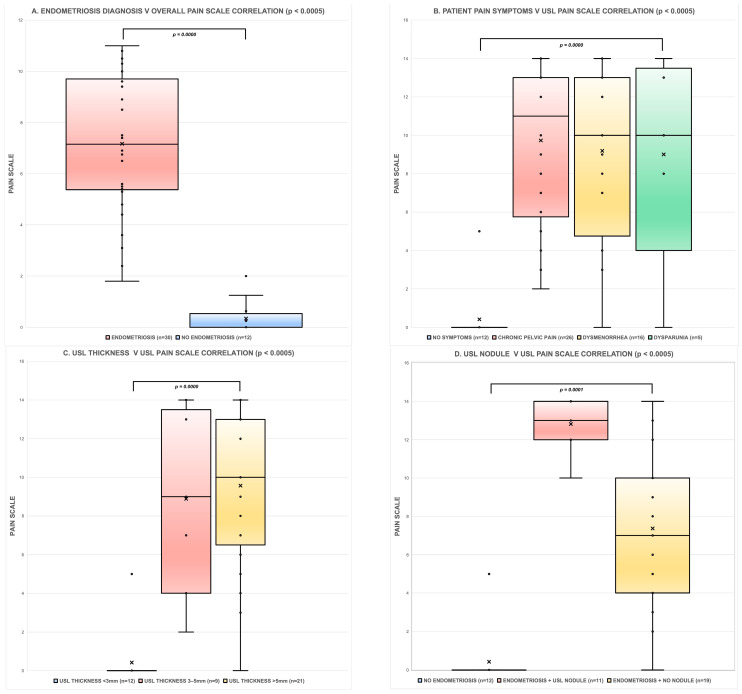
Whisker plots demonstrating the Spearman’s correlation coefficient results for endometriosis characteristics versus pain scale. These graphs show the spread of endometriosis characteristics with patient SST pain scores on TVS (where X = mean score) with statistical significance (where all *p* < 0.0005), as represented in Table 3. (**A**) Correlation of endometriosis diagnosis (surgically or clinically by referring gynecologist) and pain; (**B**) correlation of patient clinical symptoms (chronic pelvic pain, dysmenorrhea, and dyspareunia) and pain; (**C**) correlation of USL thickness and pain; and (**D**) correlation of USL nodules (endometriosis identified including a focal USL nodule and endometriosis identified with no USL nodule seen) and pain.

Results showed (Table 3) a strong correlation with pain scale score: *r* = 0.74 for clinical symptoms (chronic pelvic pain, dysmenorrhea, and dyspareunia); *r* = 0.78 for a positive or negative endometriosis diagnosis; *r* = 0.74 for USL thickness vs. pain score; and *r* = 0.70 when USL nodules were identified, all *p* < 0.0005. The graphs seen in Figure 2 visually demonstrate the correlations described.

### 3.3. Pain Descriptors for TVS Diagnosis Relationship

**Figure 3:**
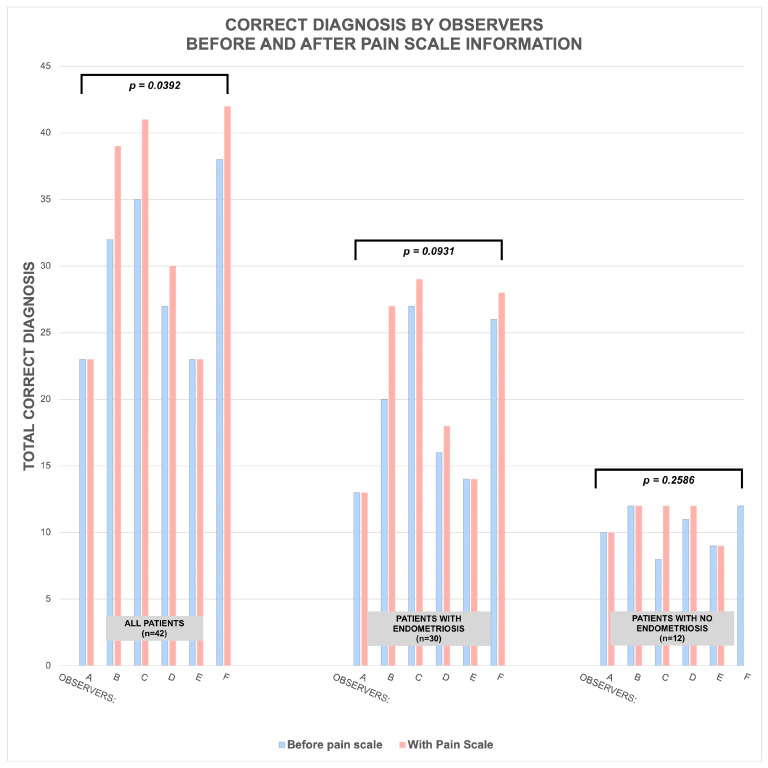
Graph representation of relationship of diagnostic scores of professional observers before and after using pain scale across the six observers, as shown in Table 4.

The paired *t*-tests showed that overall, the observers demonstrated a higher ability to correctly identify endometriosis with the pain scale information (33 ± 8.83) as opposed to not having the pain scale information (29.67 ± 6.31); a statistically significant change of 3.33 (95% CI, −6.42 to −0.24), t (5) = 2.7735, *p* = 0.0392. Table 4 shows the data spread of their diagnoses and changes, with Figure 3 graphing the results.

## 4. Discussion

This study aimed to explore the relationship between the identification of DE on TVS and pain scores on examination and its ability to assist a correct diagnosis. The mean age of the patients was 33.6 years, with all the cases of known endometriosis reporting site-specific pain on the TVS. One of the characteristics of DE is its strong association with pain [2,17], resulting in a reduction in the quality of life of its sufferers. The physical and emotional well-being of many female-born people of reproductive age is significantly affected by endometriosis pain [25,29].

While there is some academic debate regarding the association of pain symptoms and endometriosis, it is acknowledged that endometriosis has a high pain association [29]. Confliction occurs because sometimes very advanced endometriosis may not cause any symptoms [3], while in contrast, small deposits can cause severe pain [3]. However, for those individuals who have severe pain symptoms, the prevalence of DE is high [3], with this study further demonstrating that the patients with known endometriosis had significantly higher overall SF-MPQ total pain scores (7.2 ± 0.49) compared to the patients with no history of endometriosis (0.34 ± 0.19) and that there was a strong positive correlation (*r* = 0.78).

Many studies have reported that there are associations between dysmenorrhea and the size, depth, and disease stages of DE [18,19,20]. Severe dysmenorrhea does not definitely correlate with any DE location, but it does correlate with adhesions in the Pouch of Douglas [20,22,44]. Since USLs sit in this posterior compartment with their distal section attaching to the posterior cervix at the TU [8,14,28], there is an anatomical association of DE deposits at this rectocervical location, including the USLs, POD, bowel, rectum, and adhesions to the ovary in the presence of an endometrioma at the TU, all contributing to pelvic pain [6]. This study showed that the patients with an endometriosis history had significantly higher pain scores specific to USLs (8.15 ± 0.54) compared to the patients with no history of endometriosis (0.21 ± 0.21) and that there was a strong positive correlation (*r* = 0.74) with the USL pain score when correlated with the patients’ clinical symptoms of chronic pelvic pain, dysmenorrhea, and dyspareunia.

Deep dyspareunia is also correlated with endometriotic deposits of the USLs [20,44], due to the fact that USLs contain a considerable amount of nerve tissue and that neural invasion by endometriotic lesions is correlated with the severity of pain [2,25]. During intercourse, tension on the diseased USL may trigger pain [25,44] causing those individuals to have more severe sexual dysfunction [25], with the pressure, traction, or stretching of the DE nodules exacerbating the pain symptoms. During a TVS examination, this can be replicated with direct sonopalpation, allowing SST-TVS to be quite advantageous in simultaneously visualizing any DE deposits while exacerbating patient pain symptoms, hence patient-guided replication of symptoms.

**Figure 4:**
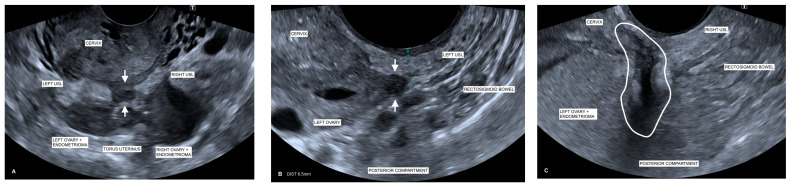
Ultrasound images correlating patient TVS and SST. (**A**) Endometriomas/medialized ovaries to USLs: Transverse TVS image shows there are bilateral endometriomas and medialization of both ovaries adherent to the USLs (right and left as labeled) at the torus uterinus. A focal endometriotic nodule (arrows) can be seen between the right and left USLs. This patient has confirmed endometriosis associated with long-standing pelvic pain. Correlating SST at this location was described as “sharp” with a total 13/15 pain score. (**B**) Thickened USL and nodule: USL DE in a woman with severe pelvic pain and dyspareunia who was confirmed to have extensive endometriosis in laparoscopy. Sagittal TVS image shows an irregular and thickened left USL (labeled with caliper measuring 6.5 mm) with adherence of the left ovary (labeled). There is an associated focal endometriotic nodule (arrows) adherent to the adjacent structures. Calipers demonstrate a 6.5 mm thickness of the left USL measured at the thickest point. Correlating SST at this location was described as “sharp” with a total 12/15 pain score. (**C**) Rectosigmoid DE adherent to USL: Sagittal TVS image demonstrating extensive deep infiltrative endometriosis. Endometriosis plaque deposits (circled) can be seen at the level of the rectosigmoid bowel, tethered to the inferior margin of the left ovary (labeled). The left ovary is medialized, with an endometrioma adherent to the torus uterinus, extending to the right USL. Correlating SST at this location was described as “sharp” with a total 10/15 pain score.

Clinically, during TVS, USLs would not be examined in isolation. However, USL examination in a routine pelvic scan is a useful starting point on its own diagnostic merit [9,12,14] to prompt further extension of the pelvic scan to look for other markers of endometriosis in the surrounding structures when a woman presents with pelvic pain [45]. Figure 4A illustrates the extensive nature of endometriosis demonstrating the association of the medialization of both ovaries with endometriomas adherent to the USLs as well as an endometriotic nodule at the TU. SST at this location was described as “sharp” with a total 13/15 pain score.

It has been previously established that the presence of a rectovaginal endometriotic nodule is always associated with pelvic pain, dysmenorrhea, and/or deep dyspareunia [2,5,21,22]. This study supported these findings, with patients reporting a particularly high degree of SST pain scores (12.8 ± 0.38), with a range of 10–14 in the patients with focal DE nodules of the USL. Findings also showed that there was a strong positive correlation with the pain score where USL nodules were identified (*r* = 0.70), as well as with USL thickness (*r* = 0.74), since USL thickness can be an indicator of DE disease [12]. Figure 4B illustrates these findings, showing an irregular and thickened left USL with adherence of the left ovary associated with a focal endometriotic nodule, with the correlating SST at this location described as “sharp” with a total 12/15 pain score. An explanation for this is that the growth of nerve fibers in endometriotic deposits is considered a mechanism of severe pain in endometriosis [17,18,46] and that there is a close histological relationship between some deep retroperitoneal endometriotic lesions and the subperitoneal nerves and that this relationship is correlated with pain [17]. It has also been demonstrated that in rectovaginal septum endometriotic nodules, a high proportion of the nerves is encapsulated in the nodular fibrosis of the deposit [2]. Endometriotic lesions are also found in close histological relationship with the nerves of the colon, even at a distance from the palpated area, and seem to infiltrate the large bowel with tenderness around the nerves [17]. In essence, a characteristic of DE nodules is that they are “pain triggers” [44]. Figure 4C visualizes this with rectosigmoid bowel DE adherent to the inferior margin of the ovarian endometrioma, extending to the right USL. Correlating SST at this location was described as “sharp” with a total 10/15 pain score.

There is clear evidence that the IDEA [8] guidelines have improved reproducible TVS diagnosis of DE in both specialist and general settings [9]. The IDEA guidelines [8] also involve checking for ‘soft markers’ such as site-specific tenderness, including over the USLs. Further studies have reported that SST on probe pressure can assist nodule identification [47]. When attention is given to site tenderness during examination, a high specificity of 94% is achieved in the detection of DE [36], with the results of this study further suggesting tenderness-directed scanning as a useful component of the ultrasound technique [12,31]. This hybrid approach of anatomical changes and pain identification follows clinical advice from Chapron et al., where “detecting a nodule during the vaginal examination by attention to subtle signs such as asymmetric uterosacral ligaments appearing irregular, hardened, and taut with the essential sign is that firm palpation of these lesions gives rise to pain identical to that of the deep dyspareunia that the patient describes” [24]. In this study, when the professional observers were given the extra SST-TVS information for each patient, there was a higher ability to correctly identify endometriosis with the pain scale information (33 ± 8.83), as opposed to not having the pain scale information (29.67 ± 6.31); a statistically significant change of 3.33 (95% CI, −6.42 to −0.24), hence indicating that a pain scale can potentially be used to improve diagnosis of endometriosis. However, there were some limitations with our results when dividing the 42 participants into those with DE and those without. While the results further demonstrated a higher ability to diagnose DE, the results were not statistically significant, with the pain scale data showing some promise, although they did not show any significant difference. An explanation for this is that the pelvic scan itself was very thorough, and the observers were familiar with the protocols and the criteria for diagnosis on the basis of pelvic scans alone.

Although a detailed pelvic scan can diagnose endometriosis, SST and pain scoring can help prompt further investigation in general settings where people are not trained to perform endo scans, which would be a practical application.

Regardless, the findings are encouraging in that there is a benefit in incorporating the patient’s pain response when considering a DE diagnosis, broadening ultrasound imaging interpretation to consider clinical elements, as all areas should be examined with consideration to each other when performing TVS for DE. This includes listening to the patient to consider SST to guide how to extend the scan to investigate for DE thoroughly.

## 5. Strengths and Limitations

There are some limitations to this study that need acknowledgment. Firstly, the observers did not perform the scan in real time as this was not feasible. Also, given the nature of the pain scale, the responses were based on a self-report, and since the perception of pain is widely variable and subjective, it was not possible to confirm. Secondly, in some cases, USL DE may have been associated with other endometriotic lesions (i.e., vagina or anterior rectal wall), which have been correlated with the presence of dyspareunia. However, this study was not designed to correlate the presence of dyspareunia with the anatomical location of endometriotic lesions. Thirdly, the results apply only to patients who had pain symptoms and therefore do not include the population of people with endometriosis who are non-symptomatic or those with endometriosis-associated pain who are managed without imaging or without seeking medical assistance. Furthermore, our study outcomes were limited to the sample size, which may not accurately represent the target population and may limit generalizability. The published Australian National Women’s Health Survey [48] reports the prevalence of chronic pelvic pain in the general population to be approximately 25–30%.

Future studies ideally need larger numbers to obtain a true representation of pain in the population. And fourthly, our study population is limited to reflect a private general imaging setting in Australia; thus, future studies with more representation are needed.

Despite these limitations, our study has several strengths, including its novelty in assessing the ultrasound relationship between site-specific tenderness and pain-related symptoms for endometriosis and the uterosacral ligaments. Other strengths include the prospective approach, blinding examiners to each other’s findings, a multidisciplinary approach, and that all assessments were performed at a single center and the images were collected by one sonographer using real-time ultrasound examinations. The reliability of our study is further strengthened by the scans conducted using a rigorous best-practice protocol. The present study provides a foundation for future research to explore the role of SST in the ultrasound diagnosis of endometriosis.

## 6. Conclusions

Individuals experiencing pelvic pain associated with endometriosis routinely have their pain discounted. This leads to considerable delays in diagnosis, resulting in years of physical suffering and associated health anxiety. This study demonstrated that patients with an endometriosis history have significantly higher pain scores on TVS compared to patients with no history of endometriosis. There was also a strong positive correlation with pain score when correlated with clinical symptoms (chronic pelvic pain, dysmenorrhea, and dyspareunia), USL thickness, and where USL nodules were identified. The findings indicate that there is value in this approach, with patient SST-TVS pain scores having a strong correlation to the ultrasound image criteria and hence, medical practitioners should be paying attention to pain symptoms experienced by patients. Listening to patients’ symptoms and incorporating these into the diagnosis of TVS could help earlier endometriosis diagnosis as well as overcome situations where patients feel that their concerns are dismissed. Inclusion of SST into TVS shows promise in the diagnosis of endometriosis. However, this finding needs to be interpreted with caution, as it was not statistically significant. Education about TVS for endometriosis, including understanding incorporating SST as a pain scale, can be of benefit, especially in clinics where endometriosis diagnostic skills are limited.

## Figures and Tables

**Table 1:** Patient characteristics and findings of TVS of the study cohort (*n* = 42).

	All Patients (*n* = 42)		with DE (*n* = 30)		No DE (*n* = 12)	
Characteristics	*n*	%	*n*	%	*n*	%
**Patient Age** (Range 18–54, Mean 33.6)						
<19	3	7.1	2	6.7	1	8.3
20–29	11	26.2	7	23.3	4	33.3
30–39	17	40.5	13	43.3	4	33.3
40–49	7	16.6	6	14.3	1	8.3
>50	4	9.5	2	6.7	2	16.6
**Diagnosis**	**30**	**71**	**30**	**71**	**12**	**28.6**
DE Diagnosed Laparoscopy	10	24	10	23.8	-	-
DE Diagnosed Clinically	7	17	20	16.7	-	-
DE Clinically Investigated	13	31	13	31	-	-
**Indication for TVS**						
Chronic Pelvic Pain	26	61.9	26	61.9	-	-
Dysmenorrhea	16	38.1	16	38.1	-	-
Infertility	6	14.3	6	14.3	-	-
Dyspareunia	5	11.9	5	11.9	-	-
Other	12	29	-	-	12	100
**Findings of endometriosis on TVS**						
Endometriosis present	30	71	30	100	0	0
Endometrioma	14	33.3	14	46.7	-	-
Obliterated POD	4	9.5	4	13.3	-	-
Adherent Ovaries	21	50	21	70.0	-	-
Medialised Ovaries	9	21.4	9	30.0	-	-
Retroverted or Rotated Uterus	11	26.2	11	36.7	-	-
Adenomyosis	19	45.2	19	63.3	-	-
Total Focal Endometriotic Lesions	21	50	21	70.0	-	-
***Types of Endometriotic Lesions:***						
Rectosigmoid DE	7	16.6	7	23.3	-	-
Rectocervical DE	9	21.4	9	30.0	-	-
USL DE (nodule)	12	28.6	12	40.0	-	-
Vaginal DE	0	0.0	0	0.0	-	-
Bladder DE	0	0.0	0	0.0	-	-
Uterine Serosal Plaques	2	4.8	2	6.7	-	-
LSCS Scar Endometriosis	1	2.4	1	3.3	-	-

KEY: (TVS) transvaginal ultrasound, (POD) Pouch of Douglas, (DE) Deep Endometriosis, (LSCS) Lower section Cesarean section. * “Other” indications for TVS include: bloating, polycystic ovarian syndrome, follow-up for endometrial polyp or hyperplasia, history of ovarian tumor, intrauterine contraception device location, and unexplained weight loss.

**Table 2:** Mean differences in pain scores and relationship of TVS and SST pain scores of patients with and without endometriosis.

Regions Where SST Was Tested During TVS	Frequency of Pain Observed			Short-Form McGill Pain Score. Mean ± SD (Range)			Correlation of Pain Scale for DE Versus No DE	
	All (*n* = 42)	with DE (*n* = 30)	No DE (*n* = 12)	All (*n* = 42)	with DE (*n* = 30)	No DE (*n* = 12)	t-Statistic (df)	*p*-Value
USL	29	28	1	5.9 ± 0.55 (0–14)	8.15 ± 0.54 (0–14)	0.21 ± 0.21 (0–5)	9.16 * (82)	0.0000
USL nodule (*n* = 11)	11	11	0	-	No Nodule (*n* = 19) 7.4 ± 0.92 (0–14) USL Nodule (*n* = 11) 12.8 ± 0.38 (10–14)	-	4.34 * (28)	0.0002
Ovary	33	30	3	6.4 ± 0.53 (0–15)	8.7 ± 0.47 (0–15)	0.63 ± 0.29 (0–8)	10.30 * (82)	0.0000
Post Fornix	31	29	2	6.5 ± 0.76 (0–13)	8.7 ± 0.70 (0–13)	1.1 ± 0.75 (0–8)	6.25 * (40)	0.0000
POD	21	21	0	4.3 ± 0.76 (0–13)	6.0 ± 0.89 (0–13)	0, 0 (0)	4.24 * (40)	0.0001
Bladder	8	8	0	1.2 ± 0.42 (0–10)	1.6 ± 0.57 (0–10)	0, 0 (0)	1.72 (40)	0.0926
Bowel nodule (*n* = 16)	16	16	0	-	No Nodule (*n* = 14) 0, 0 (0) Bowel Nodule (*n* = 16) 13.7 ± 0.28 (10–15)	-	44.89 * (28)	0.0000
All Areas	34	30	4	5.2 ± 0.59 (0–10.5)	7.2 ± 0.49 (1.8–11)	0.34 ± 0.19, (0–2)	8.87 * (40)	0.0000

KEY: (TVS) transvaginal ultrasound, (SST) site-specific tenderness, (SD) Standard Deviation, (*n*) sample size, (DE) Deep Endometriosis, (USL) uterosacral ligament, (POD) Pouch of Douglas, (*) statistically significant.

**Table 3:** Correlation of endometriosis characteristics vs. pain scale score.

Characteristic	(*n*)	Spearman’s Correlation (*r*)	*p*-Value	Interpretation
Endometriosis diagnosis vs. Overall pain scale score	42	0.78 *	0.0000	Strong Correlation
Patient pain symptoms vs. USL pain scale score	42	0.74 *	0.0000	Strong Correlation
USL thickness vs. USL pain scale score	42	0.74 *	0.0000	Strong Correlation
USL nodule vs. USL pain score	30	0.70 *	0.0001	Strong Correlation

KEY: (*r*) Spearman’s correlation, (*n*) sample size, (USL) uterosacral ligament, (*) statistically significant.

**Table 4:** Relationship scores between diagnosis by professional observers as a collective group and individually, based before and after using pain scale across the six observers.

Variables	Before Pain Score Information Mean (DF)	After Pain Score Information Mean (DF)	Score Difference Mean (95% CI)	t-Statistic (df)	*p*-Value
**All Observers**					
All Patients (*n* = 42)	29.67 (6.31)	33.00 (8.83)	3.33 (−6.42, −0.24)	2.77 * (5)	0.0392
Patients With DE (*n* = 30)	19.33 (6.06)	21.50 (7.34)	2.17 (−4.86, 0.52)	2.071 (5)	0.0931
Patients With No DE (*n* = 12)	10.33 (1.63)	11.17 (1.33)	2.17 (−2.51, 0.85)	1.27 (5)	0.2586
**Individual Observers**					
Observer A	15.3	15.3	0	-	-
Observer B	21.3	26	4.7	2.00 (2)	0.1835
Observer C	23.3	27.3	4	3.46 (2)	0.0742
Observer D	18	20	2	3.46 (2)	0.0742
Observer E	15.3	15.3	0	-	-
Observer F	25.3	27.3	2	1.7321 (2)	0.2254

KEY: (CI) confidence interval, (*n*) sample size, (DF) degrees of freedom, (DE) Deep Endometriosis, (*) statistically significant.

## Data Availability

The data presented in this study are available on request from the corresponding author due to patient confidentiality restrictions.
